# Family planning in the Atwima Nwabiagya North District: A follow-up on attitudes and practices

**DOI:** 10.4102/jphia.v17i1.1285

**Published:** 2026-02-04

**Authors:** Alexa J. Henrie, Mariam Atobiloye, Bryan Radmall, Quincy N. Sorensen, Andy Yanagihara, Daniel Ansong, Eric Sarpong, Lowell S. Benson, Ty Dickerson

**Affiliations:** 1 Department of Family and Preventive Medicine, University of Utah School of Medicine, Salt Lake City, United States of America; 2 Department of Family and Preventive Medicine, University of Utah, Salt Lake City, United States of America; 3 Komfo Anokye Teaching Hospital, Kwame Nkrumah University of Science and Technology, Kumasi, Ghana; 4 Atwima Nwabiagya North Health District, Kumasi, Ghana; 5 Department of Public Health and Infectious Diseases, University of Utah School of Medicine, Salt Lake City, United States of America; 6 Department of Pediatrics, University of Utah School of Medicine, Salt Lake City, United States of America

**Keywords:** family planning, contraceptive use, global health, follow-up study, pregnancy intentions

## Abstract

**Background:**

In 2010, the Barekuma Collaborative Community Development Project demonstrated concerning evidence of unmet family planning needs in the Barkese sub-district in the Ashanti region of Ghana. In 2023, Ghana Health Services requested the study be repeated to understand how the prevalence of abortions, family planning perceptions and methods used by women in this region had changed since 2010.

**Aim:**

The aim of this study is to understand how the region’s attitudes and behaviours associated with contraception have changed since 2010.

**Setting:**

Survey participants were from 10 communities in the Atwima Nwabiagya North District.

**Methods:**

One hundred and ninety-eight women participated in a survey with questions adapted from the 2008 Ghana Demographic and Health Survey. Once responses were collected, descriptive analysis was performed to determine trends in the use of contraceptives and reasons against the use of family planning.

**Results:**

Seventy-six point 5 per cent of women had used at least one family planning method. Emergency contraception was the most used method ever. One hundred and forty-seven had reproductive potential. Of these women, 47.6% were currently using a method to prevent pregnancy, most commonly the rhythm (calendar) method. When asked about their latest pregnancy, 65.7% were not trying to get pregnant at that time. Fear of side effects was a common reason for avoiding use of contraceptives.

**Conclusion:**

Compared to the 2010 study, emergency contraception replaced oral contraceptives as the most ever used method. Fewer women were currently using a method to prevent pregnancy, and unintended pregnancies occurred more frequently. The fear of adverse side effects identified in the 2010 study continues to be a major barrier today.

**Contribution:**

This study affirms the persistence of unplanned pregnancies and limited knowledge of contraceptive side effects, indicating the need for comprehensive family planning education.

## Introduction

In 2014, the West African nation of Ghana completed a Demographic and Health Survey (GDHS), providing a collection of data to monitor the health of Ghanaian populations. The survey found that there was an increase in the usage of family planning methods from 13% to 27% since 1988.^1^ However, the survey also found concerning evidence of unmet family planning needs.^1^

Additionally, the GDHS cited that only 27% of married women in Ghana reported using any modern or traditional method of contraception, despite three in 10 married women reporting the need to space out or halt childbearing.^1^ For unmarried, sexually active women aged 15–49 years, only 32% used any type of contraception for family planning.^1^ Reporting of contraception use was positively associated with the education and economic status of the respondent.^1^

In 2016, a follow-up study to this survey found that there was little change to these statistics.^2^ Additionally, the study proposed that there were a multitude of reasons behind the sense of aversion to contraception by Ghanaian women, including partner opinion, religion and, above all, fear of possible adverse side effects associated with contraceptive use.

In 2010, the Barekuma Collaborative Community Development Project (BCCDP), composed of collaborators from the Komfo Anokye Teaching Hospital (KATH), Kwame Nkrumah University of Science and Technology (KNUST) and the University of Utah School of Medicine, conducted community-based participatory research regarding knowledge, attitudes and practices related to family planning in the Barekese sub-district (now part of the Atwima Nwabiagya North District) in the Ashanti Region of Ghana. The 2010 study explored (1) specific reasons for not using various methods of contraception; (2) what methods women had heard of and used; (3) perceptions and attitudes towards family planning; and (4) potential interest in continued family planning education. The study concluded that there were a substantial number of women with misinformation regarding the usage of a widely available synthetic progestin hormone tablet, believing it to be a form of emergency contraception. It was also found that nearly one-third of women avoided modern contraception methods because of fear of adverse side effects. Urgent intervention was requested to promote community education through trained community health workers.

In 2023, local Ghana Health Services leadership requested the 2010 study be repeated for additional evidence to inform potential interventions related to family planning and contraception programmes in the Atwima Nwabiagya North District. The focus was to understand how attitudes and perceptions of contraception had changed in the region since 2010, specifically, the prevalence of abortions, pregnancy intentions, family planning perceptions and receptions and methods used by Ghanaian women in this region.

## Research methods and design

### Study design

This study was conducted from 05 July 2023 to 13 July 2023 and was a replication of a cross-sectional study conducted in 2010.^3^ Questions from the 2008 Ghana Demographic and Health Survey (DHS) were adapted and digitised to Kobocollect software. The 2008 Ghana DHS was selected, as this was the survey utilised in the 2010 study. Questions focused on demographic information, reproductive health and history, family planning usage and recognition and access to care, among other topics.

### Setting

Surveys were administered in 10 communities in the Atwima Nwabiagya North District of the Ashanti Region of Ghana, West Africa.

### Study population and sampling strategy

Women aged 14 years and older were eligible to complete the survey. As this was a replication of the 2010 study, eligibility was kept as close to the original study as possible. The age inclusion criteria was expanded to include younger women. Eligibility was not restricted in any other way. Survey participants were identified by convenience sampling. Participants were located throughout the communities, generally in their households or at their workplaces. The number of women approached about the survey, the response rate and the number of ineligible persons were not recorded.

### Data collection

Local community health nurses trained in data collection and survey administration acted as translators between research assistants and survey participants. Upon introduction, the survey was briefly introduced to each potential participant, and eligibility was ascertained. Eligible participants were invited to respond to the survey, and, upon acceptance, informed consent was obtained. As children under the age of 18 years cannot provide informed consent, informed consent from a parent or guardian was obtained, and then assent (agreement from an individual unable to provide informed consent) from the participant herself was obtained. The interview took approximately 1 h to complete for each respondent. Kobocollect, data collection software available online and on the Apple and Google app stores for free, was used for data entry and storage.

### Data analysis

Once responses were collected, descriptive statistical analysis was performed to determine demographic distribution of participants, common family planning perceptions and opinions, knowledge, ever and current use of contraceptives and reasons against use of family planning. Confidence intervals for all proportional data were calculated using normal approximation and were truncated at 0% – 100%. Proportions of respondents who recognised specific family planning methods were determined by counting the number of ‘recognitions’ coded onto Kobocollect for each contraceptive method. The number of recognitions was then divided by the total number of respondents. The ever and current use of family planning methods was determined by counting the number of respondents who said they had used a specific family planning method, divided by the number of respondents who were at reproductive potential at the time of the interview (based on menstruation status). Pregnancy intentions were elucidated by asking women who were either pregnant at the time of the interview or who had been pregnant at least once in the past whether they wanted to become pregnant at the time of conception, if they had wanted to wait until later to conceive or if they did not want any more children at all when they learned of their pregnancy. Responses to each category were counted and then divided by the number of respondents who had had at least one pregnancy in their lifetime. For open-ended responses, textual data were coded and categorised.

### Ethical considerations

An application for full ethical approval was made to the Institutional Review Board at a public university in the Intermountain West, United States, and ethics consent was received on 6/27/2023. The ethics approval number is IRB_00167941. All procedures performed were in accordance with the ethical standards of the Internal Review Board at the public university in the Intermountain West, United States, and with the 1964 Helsinki Declaration and its later amendments or comparable ethical standards. No identifying information was collected from the participants, and survey responses were assigned unique identifiers. Collected data were only accessible by the study team through password-protected accounts. Written informed consent was obtained from all individual participants involved in the study. For participants under 18, written informed consent was obtained from their parents or guardians.

## Results

### Demographics of participants

The demographics of the participants who completed the survey are presented in Table 1. One hundred ninety-eight women aged 14–75 years from the Atwima Nwabiagya North District of Kumasi, in the Ashanti region of Ghana, participated in this study. These participants were from 10 communities: Abira, Achina, Adankwame, Aninkroma, Barekese, Barekroma, Fufuo, Kumi, Maban and Worapong. The mean age of the participants was 31.4 years. When their levels of education were assessed, four (2.0%) of the women attended higher ed/post-secondary school, 44 (22.2%) attended senior secondary school, most (*n* = 102; 51.5%) attended junior secondary school and 19 (9.6%) attended primary school. Furthermore, 163 (82.7%) of those who responded identified as Christians, encompassing several dominations such as Pentecostal, Methodist, Presbyterian and Catholic, while 30 (15.2%) identified as Muslims. A small proportion (*n* = 4; 2.0%) reported no affiliation with any organised religion. Regarding marital status, 77 (40.7%) of the respondents were married at the time the survey was conducted, 41 (21.7%) were unmarried but living with a man and 71 (37.6%) were not in a union or living with a man at the time of the survey (Table 1).

**TABLE 1:** Demographic characteristics of 14–75-year-old respondents included in the study (*n* = 195).

Variable	*n*	% of respondents	95% CI
**Age (years)**			
14–23	73	37.4	30.6–44.2
24–33	48	24.6	18.6–30.6
34–43	46	23.6	17.6–29.6
44–53	16	8.20	4.3–12.1
54–63	5	2.60	0.4–4.8
64–73	4	2.10	0.1–4.1
74–75	3	1.50	0.0–3.2
Total	195	-	-
**Highest level of education**			
Primary school	19	9.60	5.5–13.7
Junior secondary school	102	51.5	44.5–58.5
Senior secondary school	44	22.2	16.4–28.0
Post-secondary/higher education	4	2.00	0.0–4.0
No response	29	14.7	9.8–19.6
Total	198	-	-
**Religion**			
Christian	163	82.7	77.4–88.0
Muslim	30	15.2	10.2–20.2
No religion	4	2.00	0.0–4.0
Total	197	-	-
**Are you currently living with a man as if married?**			
Yes, currently married	77	40.7	33.7–47.7
Yes, living with a man	41	21.7	15.8–27.6
No, not in union	71	37.6	30.7–44.5
Total	189	-	-

Note: Age (years) – mean = 31.4; median =27; range = 14–75.

CI, confidence interval.

### Family planning perception and opinion

Participants’ perceptions of family planning were assessed through five questions detailed in Table 2 and Table 3. Most of the participants who responded (*n* = 128; 67.4%) disagreed with the statement ‘contraception is a woman’s business, and a man should not have to worry about it’, whereas 58 (30.5%) of them agreed. In a similar manner, when the participants were asked about the statement, ‘women who use contraception may become promiscuous’, almost two-thirds (*n* = 113; 59.5%) disagreed and 71 (37.4%) agreed. Conversely, agreement was higher for statements underscoring the risks of having too many children and the benefits of smaller families for children’s success. 135 (71.1%) agreed that ‘having too many children may be dangerous for a woman’, 162 (85.3%) agreed that ‘it is better not to have more children than we can afford’ and 154 (81.1%) agreed with the statement that read, ‘children in smaller families are more likely to succeed’. Additionally, when asked about their opinions on decision-making when it comes to contraceptive use, 42 (22.2%) believed it was ‘mainly your decision’. In contrast, a significant majority (*n* = 134; 70.9%) thought that it should be a joint decision between themselves and their partners.

**TABLE 2:** The ‘opinions’ Ghanaian women in the Atwima Nwabiagya North District of Kumasi have about family planning.

Your opinion on using contraception	*n*	% of respondents	95% CI
Mainly your decision	42	22.2	16.3–28.1
Joint decision	134	70.9	64.4–77.4
Other	13	6.90	3.3–10.5

**Total**	**189**	-	-

CI, confidence interval.

**TABLE 3:** The ‘perceptions’ Ghanaian women in the Atwima Nwabiagya North District of Kumasi have about family planning.

Family planning perception	Agree		Disagree		Neutral or no opinion	
	%	95% CI	%	95% CI	%	95% CI
Contraception is a woman’s business, and a man should not have to worry about it	30.5	24.0–37.1	67.4	60.7–74.0	2.1	0.1–4.1
Women who use contraception may become promiscuous	37.4	30.5–44.2	59.5	52.5–66.5	3.7	1.0–6.4
Having too many children may be dangerous for a woman	71.1	64.6–77.5	22.6	16.7–28.6	6.3	2.9–9.8
It is better not to have more children than we can afford	85.3	80.2–90.3	14.2	9.2–19.2	1.1	0.0–2.5
Children in smaller families are more likely to succeed	81.1	75.5–86.6	13.7	8.8–18.6	4.7	1.7–7.8

CI, confidence interval.

### Family planning knowledge

The participants’ baseline knowledge of family planning was also assessed, as depicted in Table 4. Only 69 (35.2%) respondents were aware of the counting method to monitor fertility. Likewise, 60 (30.6%) fully understood and had heard about using body signs to monitor fertility. Despite this, a notable interest was shown in learning these methods, with 59.3% (*n* = 112) and 62.4% (*n* = 118) expressing a willingness to learn about the counting and body signs methods, respectively. Approximately, half of the respondents (*n* = 102; 51.8%) mentioned that they had visited a healthcare facility within the past year, and of these respondents, 49 (48.0%) received counselling on family planning methods during these visits. However, the results show that 22 (64.7%) of the respondents were not told about the side effects of their preferred family planning method when they obtained it last, and 18 (81.8%) had never been told about these potential side effects of contraception by a healthcare worker.

**TABLE 4:** Survey responses of the knowledge and reception of family planning from the women of the Atwima Nwabiagya North District of Kumasi.

Variable	*n*	% of respondents	95% CI
**Have you heard of a counting method to monitor your fertility?**			
No	127	64.8	58.1–71.5
Yes	69	35.2	28.5–41.0
**Would you be interested in learning and using a method to avoid pregnancy that helps you count the days of fertility and infertility with beads on a string?**			
No	77	40.7	33.7–47.7
Yes	112	59.3	52.3–66.2
**Have you heard of body signs as a method to monitor your fertility?**			
No	136	69.4	63.0–75.8
Yes	60	30.6	24.2–37.0
**Would you be interested in learning and using a method to avoid pregnancy that teaches you how to recognise the body’s signs of fertility and infertility?**			
No	71	35.6	28.7–42.5
Yes	118	62.4	55.5–69.3
**In the last 12 months, were you visited by a fieldworker who talked to you about family planning?**			
No	174	88.3	83.8–92.8
Yes	23	11.7	7.2–16.2
**Did the fieldworker discuss family planning methods with you?**			
No	2	8.70	0.0–20.3
Yes	21	91.3	79.7–100.0
**In the last 12 months, have you visited a health facility for care for yourself (or your children)?**			
No	95	48.2	41.0–55.4
Yes	102	51.8	44.6–59.0
**Did any staff member at the health facility speak to you about family planning methods?**			
No	53	52.0	42.5–61.5
Yes	49	48.0	38.5–57.5
**When you obtained your last family planning method, were you told about the side effects of this method?**			
No	22	64.7	51.0–78.4
Yes	12	35.3	21.6–49.0
**Were you ever told by a health worker about side effects or problems you might have with the method?**			
No	18	81.8	66.8–96.8
Yes	4	18.2	3.2–33.2

CI, confidence interval.

### Family planning recognition, use and preferences

Table 5 describes the recognition of, ever use and current use of and future preferences of family planning methods among women in our study. Most women recognised at least one family planning method (98.5%), and 76.5% had used at least one method. Women recognised injectables (86.2%), male condoms (84.7%) and daily oral contraceptives (80.1%) most frequently. Emergency contraception (32.7%), withdrawal (27.6%), injectables (25.5%) and daily oral contraceptives (25.5%) were the most cited ever-used methods. Male sterilisation, the diaphragm and foam/jelly had the lowest rates of recognition, as well as ever and current use. 147 women were menstruating and therefore had reproductive potential at the time of the interview. Of these women, 47.6% were currently using a method to delay or avoid pregnancy. The most popular current method among women at risk for pregnancy was the rhythm (calendar) method (12.2%). The second most popular method was implants (10.2%). When asked about potential future usage, 62.1% of respondents stated they intend to use some form of family planning. Injectables (18.5%), implants (13.8%) and rhythm (calendar) (11.3%) were the most popular preferred future methods.

**TABLE 5:** Recognition, ever use and current use and future preferences of family planning methods among 14–75-year-old Ghanaian women in the Atwima Nwabiagya North District included in the study (*N* = 196).

Family planning method	Recognises		95% CI	Ever use		95% CI	Current use		95% CI	Future preferences		95% CI
	*n*	%		*n*	%		*n*	%		*n*	%	
Female sterilisation	137	69.9	63.5, 76.3	6	3.1	0.7, 5.5	6	4.1	0.9, 7.3	8	4.1	1.3, 6.9
Male sterilisation	38	19.4	13.9, 24.9	0	0.0	-	0	0.0	-	0	0.0	-
Pill	157	80.1	74.5, 85.7	50	25.5	19.4, 31.6	13	8.8	4.2, 13.4	15	7.7	4.0, 11.4
Intrauterine device	83	42.3	35.4, 49.2	4	2.0	-	0	0.0, 4.0	-	3	1.5	0.0, 3.2
Injectable	169	86.2	81.4, 91.0	50	25.5	19.4, 31.6	8	5.4	1.7, 9.1	36	18.5	13.1, 23.9
Implant	154	78.6	72.9, 84.3	26	13.3	8.5, 18.1	15	10.2	5.3, 15.1	27	13.8	9.0, 18.6
Condom	166	84.7	79.7, 89.7	19	9.7	5.6, 13.8	1	0.7	0.0, 2.0	7	3.6	1.0, 6.2
Female condom	115	58.7	51.8, 65.6	1	0.5	0.0, 1.5	0	0.0	-	1	0.5	0.0–1.5
Diaphragm	9	4.6	1.7, 7.5	0	0.0	-	0	0.0	-	0	0.0	-
Foam or jelly	4	2.0	0.0, 4.0	0	0.0	-	0	0.0	-	0	0.0	-
Rhythm (calendar)	119	60.7	53.9, 67.5	45	23.0	17.1, 28.9	18	12.2	6.9, 17.5	22	11.3	6.9, 15.7
Withdrawal	131	66.8	60.2, 73.4	54	27.6	21.3, 33.9	3	2.0	0.0, 4.3	11	5.6	2.4, 8.8
Lactational amenorrhea method (LAM)	100	51.0	44.0, 58.0	42	21.4	15.7, 27.1	4	2.7	0.1, 5.3	1	0.5	0.0, 1.5
Emergency contraception	125	63.8	57.1, 70.5	64	32.7	26.1, 39.3	N/A	N/A	-	6	3.1	0.7, 5.5

**Total**	**193**	**98.5**	**96.8, 100**	**150**	**76.5**	**70.6, 82.4**	**70**	**47.6**	**39.5, 55.7**	**121**	**62.1**	**55.3, 68.9**

Note: Current use of family planning methods was only asked of women capable of menstruation at the time of the interview (*n* = 147).

CI, confidence interval.

### Abortion and pregnancy intentions

Nine point 6 per cent of women had had at least one prior abortion at the time of the survey administration. When asked about their latest or current pregnancy, 34.3% of women were trying to get pregnant at that time, 35.7% wanted to wait until later and 30.0% of women did not want any more children when they learned of their pregnancy (Table 6).

**TABLE 6:** Abortions and pregnancy intentions of women who had at least one pregnancy at the time of the interview (*n* = 140).

Last or current pregnancy intention	*n*	%	95% CI
Did not want any more children	42	30.0	22.4–37.6
Wanted to become pregnant then	48	34.3	26.4–42.2
Wanted to wait until later	50	35.7	27.8–43.6
Women who have had an abortion	19	9.6	5.5–13.7

CI, confidence interval.

### Reasons for not using family planning methods

We asked study participants open-ended questions concerning why they would not choose to use specific forms of contraception. Table 7 summarises the top three reasons why women stated they would not use specific forms of contraception. The most common reason expressed for not using any method of contraception was having no need for contraception and/or already using some form of contraception. Fear of potential complications and side effects was the top reason stated for not wanting to use the oral pill, intrauterine devices (IUDs), injectables and implants. The method being unknown or unavailable was the top response for not using male sterilisation, diaphragms, foam or jelly and the rhythm (calendar) method. Not preferring or liking the method was the most common reason for not wanting to use male or female condoms, the withdrawal method and emergency contraception. The top stated reason for not wanting to use female sterilisation was wanting to become pregnant again or wanting more children. The leading reason for not using lactational amenorrhoea was the method being ineffective and/or not working for the respondent.

**TABLE 7:** The top three reasons Ghanaian women in the Barekese sub-district said they would not use various family planning methods with the number and percent of respondents who gave that response.

Family planning method	Top reason	*n*	%	2nd reason	*n*	%	3rd reason	*n*	%
Any method	Has no need for contraception/already using another form of contraception	23	33.8	Afraid of complications/side effects	14	20.6	Wants to become pregnant again/wants more children	8	11.8
Female sterilisation	Wants to become pregnant again/wants more children	33	23.7	Afraid of complications/side effects	24	17.3	(Tied) Method is unknown/unavailable and not preferred/doesn’t like method	21	15.1
Male sterilisation	Method is unknown/unavailable	37	28.7	Wants to become pregnant again/wants more children	18	14.0	Not preferred/doesn’t like method	17	13.2
Pill	Afraid of complications/side effects	30	23.6	Not preferred/doesn’t like method	24	18.9	Ineffective/won’t work for her	23	18.1
Intrauterine device	Afraid of complications/side effects	41	30.6	Method is unknown/unavailable	30	22.4	Not preferred/doesn’t like method	24	17.9
Injectable	Afraid of complications/side effects	36	32.4	Not preferred/doesn’t like method	20	18.0	Has no need for contraception/already using another form	16	14.4
Implant	Afraid of complications/side effects	49	39.5	Not preferred/doesn’t like method	29	23.4	Has no need for contraception/already using another form	17	13.7
Male condom	Not preferred/doesn’t like method	55	39.9	Method is unknown/unavailable	18	13.0	Has no need for contraception/already using another form	17	12.3
Female condom	Not preferred/doesn’t like method	44	33.6	Method is unknown/unavailable	36	27.5	Has no need for contraception/already using another form	17	13.0
Diaphragm	Method is unknown/unavailable	66	52.8	Has no need for contraception/already using another method	16	12.8	Not preferred/doesn’t like method	15	12.0
foam or jelly	Method is unknown/unavailable	68	54.4	(Tied) Has no need for contraception/already using another method and not preferred/doesn’t like method	16	12.8	Unsure/no reason	8	6.4
Rhythm (Calendar) method	Method is unknown/unavailable	25	22.7	Has no need for contraception/already using another method	20	18.2	Ineffective/won’t work for her	18	16.4
Withdrawal	Not preferred/doesn’t like method	29	27.1	Method is unknown/unavailable	20	18.7	Has no need for contraception/already using another form	17	15.9
Lactational Amenorrhea method	Ineffective/won’t work for her	37	33.3	Method is unknown/unavailable	18	16.2	Has no need for contraception/already using another form	17	15.3
Emergency contraception	Not preferred/doesn’t like method	25	21.9	(Tied) Has no need for contraception/already using another method and method is unknown/unavailable and afraid of complications/side effects	21	18.4	-	-	-

Another common reason expressed for not using specific forms of contraception was that it was not the woman’s decision. This reason was given most often referring to male sterilisation (8 respondents), male condoms (11 respondents) and the withdrawal method (10 respondents). Another common response given for all methods of contraception was the respondent being unsure or having no reason for not using that method.

## Discussion

Over a decade has passed since the pivotal study on family planning in the Barekese sub-district was conducted.^3^ It aimed to identify method-specific information regarding family planning beliefs, practices and preferences among women to further inform family planning programmes, research and policy.^3^ Reflecting on the necessity to understand the evolution of family planning practices and attitudes, our study embarked on a follow-up investigation 13 years later. Common themes across these studies include (1) educational achievement and family planning knowledge, (2) female perceptions of family planning, (3) recognition of family planning methods, uses and preferences, (4) knowledge of male and female sterilisation, (5) fear of adverse contraception side effects and (6) abortion and pregnancy intentions.

### Educational achievement and family planning knowledge

Most of the women who were surveyed were educated with a primary school education at the minimum, and the results show that there appears to be a trend between their level of education and the knowledge of contraceptives that they possess. Despite this, the unmet need for family planning remained. This observation corroborates a similar finding in a study where it was suggested that the awareness and knowledge of contraception do not translate to its use.^4^ While educational attainment is generally known to positively influence the use of modern contraceptive methods in low- and middle-income countries,^5^ a study from a Zimbabwean community showed that adolescent women with more years of schooling and higher levels of secondary education were less likely to use contraceptives.^6^ This result underscores the complexity of contraceptive awareness and indicates that factors beyond educational attainment, such as cultural or social influences, may play critical roles in shaping family planning knowledge and/or use.

### Views on family planning

Our study highlighted some of the insights into family planning among the women in the Atwima Nwabiagya North District. Although a notable minority of the respondents believed that family planning and contraception use were solely a woman’s decision, over two-thirds of the women who took the survey recognised the importance of joint decision-making. This finding is in line with their beliefs on family planning, where 67.4% of the women disagreed with the statement, ‘contraception is a woman’s business, and a man should not have to worry about it’. Furthermore, this provides a shift in perspective from the 2010 study, where more women agreed with the statement,^3^ suggesting an evolving understanding of family planning as a shared responsibility rather than a burden meant for women exclusively. This buttresses one of the conclusions from a study where ‘couple-based family planning interventions’ were encouraged to address the unmet need for family planning in a district of a sub-Saharan country.^7^ Overall, our findings highlight and reveal that women in this community now place greater emphasis on shared decision-making when it comes to their reproductive health.

In both studies, higher proportions of women agreed with the statements ‘having too many children may be dangerous for a woman’ and ‘it is better not to have more children than we can afford’. However, a larger proportion agreed with the latter statement in the 2023 study. Similar proportions in both studies agreed with the statement ‘children in smaller families are more likely to succeed’. The total fertility rate (TFR) worldwide has continued to decline, with rapid decreases in Asia and Latin America and slower but still occurring declines in sub-Saharan Africa.^8^ Exposure to mass media, cultural changes, religious factors, the spread of individualism from Western cultures and the preference for smaller family sizes compared to previous generations are among the reasons for the reduction in TFR in many countries worldwide.^9^ Specifically, Ghana has continued to face economic turmoil since 2010, as evidenced by the fall in Gross Domestic Product (GDP).^10^ This shift in thought processes regarding the number of children might mirror some of the socio-economic hardships many Ghanaian families face. It is a suggestion that further supports the idea that economic considerations are becoming more important in family planning decisions.

Attitudes regarding promiscuity in relation to contraception showed a marked difference. In the previous study, nearly two-thirds of the women concurred with the statement that ‘women who use contraception may become promiscuous’. In contrast, the current research indicated that just over one-third of women held this view. Previous studies have shown that women in parts of Ghana, as well as women in other developing nations in Africa, view contraceptive use as promiscuous.^11,12^ A 2022 Ghanaian demographic study showed that most men, particularly those aged 20–24 years, those with secondary education, married men and men living in rural areas, agreed that contraceptive use leads women to promiscuity.^13^ However, our results show that most of the women in this community now disagree with this view. This finding could aid in the reduction of stigmatisation around contraception, which can help decrease the unmet need for contraception.

### Family planning recognition, use and preferences

There are key differences in contraceptive method recognition, use and preferences since the 2010 study. For example, female sterilisation was the third most recognised form of family planning in the original study, and it is now the fifth most recognised method.^3^ Furthermore, emergency contraception replaced oral contraceptives as the most widely ever used method among women (see Table 5). This finding is consistent with a recent study in 2018 that found that emergency contraception was a common method of pregnancy prevention in young, urban women living in Accra, Ghana.^14^ Another study from 2018 indicated the presence of extensive misinformation regarding the correct usage and safety of emergency contraception within these communities.^15^ These findings suggest the need for more education regarding the role of emergency contraception within family planning, as it is both very popular and misused as a primary prevention for pregnancy. Fewer women were currently using a method to delay or avoid pregnancy than in 2010 (by 7.3%). The most popular current method among women at risk for pregnancy was the rhythm (calendar) method, whereas in 2010, it was oral contraceptives.^3^ Male sterilisation, diaphragms and foam and jelly had the lowest rates of use and recognition in both studies. When asked about potential future usage, 62.1% of respondents stated they intend to use some form of family planning, compared to 85.9% of respondents who indicated future use in 2010. Injectables were the most popular preferred future method, while in 2010, it was oral contraceptives. Overall, current use and expected use of family planning methods have decreased since the original study. Method preference has also changed, with more reliance on non-hormonal methods of contraception and a rise in popularity of implants and injectables over former hormonal methods like daily oral contraceptives. It is important to note that castor seed oil was mentioned spontaneously by 18 survey respondents (9.2%) as a common mode of contraception. Twelve women (6.1%) mentioned enemas as a form of contraception as well. Castor oil is used as a laxative, so women may have been referring to the same thing when they stated that castor oil and enemas are used for pregnancy prevention. Castor oil’s use for contraception is an ancient practice among the Rukuba women in Nigeria; they would consume a couple of seeds per year to prevent pregnancy.^16^ Furthermore, there may have been confusion defining the difference between emergency contraception and daily oral contraceptive pills. During the previous study, researchers found that 27% of women reported using ‘N-tablets’, the common name for Primolut N tablets (a pill containing five mg of synthetic progesterone called norethisterone or norethindrone), as emergency and daily contraception.^3^ At the time, no study had been conducted to determine the effectiveness of using a norethisterone-only pill for emergency contraception, and no organisation or agency had recommended its use.^3^ The 2023 follow-up study did not find that women were using ‘N-tablets’ as a primary method of contraception. However, the current use of general emergency contraception was not asked about during the survey.

### Knowledge of male and female sterilisation

Most of the women who were interviewed recognised female sterilisation (69.9%) as a family planning method. In contrast, a minority of the women recognised male sterilisation as a mode of birth control (19.4%). Furthermore, 28.7% of respondents reported that they would not choose male sterilisation for contraception because the method was unknown or unavailable to them. This contrasts with reasons given for not using female sterilisation, where only 15.1% of respondents stated that the method was unknown or unavailable. This gap in knowledge of male versus female sterilisation may simply be because all study participants were female and are, therefore, more likely to be informed about female-dependent methods of contraception. It might also be reflective of the perception reported by nearly one-third of survey participants that contraception is a woman’s business, and a man should not have to worry about it. This reinforces the need for future studies focused on the attitudes and beliefs of men in Ghana regarding family planning.

### Fear of adverse contraception side effects

Similar to the 2010 study, we found that one of the leading reasons participants stated they would not use certain methods of contraception was a fear of adverse side effects and complications. While our survey did not explicitly ask respondents to state what side effects they were most worried about, many women provided specific responses. Commonly mentioned side effects included hypertension, dizziness, weight gain, heart palpitations, permanent loss of fertility and loss of regular menses. The latter concern may suggest inadequate education surrounding some forms of contraception, such as the pill, which primarily prevents pregnancy by altering the menstrual cycle. While many of the other side effects mentioned by participants are, in fact, known side effects caused by various forms of contraception,^17^ it is unclear if women were accurately informed as to the frequency and severity of those side effects.

It is important to consider what sources women in Ghana utilise to learn about contraceptives and their side effect profiles. While many respondents reported they had personally experienced adverse effects when using a contraceptive, many others stated that they were afraid of one or more methods of contraception because of the adverse side effects a friend or family member told them about. This suggests that many women in Ghana depend upon close contacts to inform their decisions relating to contraceptive use as opposed to seeking out the advice of medical experts. Further information is needed regarding what sources women in Ghana trust most regarding their reproductive health and particularly regarding the benefits and side effects of contraceptives.

### Abortion and pregnancy intentions

In the previous study, 20% of respondents had personally experienced at least one abortion at the time of the survey administration.^3^ This is 10.4% more than women who had at least one abortion in our 2023 study (see Table 6). However, there was a 10.6% decrease in women who had timely pregnancies in 2023, suggesting that planned pregnancies happened less frequently in the most recent study. Furthermore, 30% of women did not want any more children when they conceived their most recent pregnancy, which is a 17% increase in unwanted pregnancies since 2010.^3^ Overall, this suggests that unintended pregnancies are occurring more frequently now than they did a decade ago.

### Strengths and limitations

Our study benefited from high respondent participation rates, and the questionnaire was modelled on the validated questions from the 2008 Ghana Demographic and Health Survey. Similar to the 2010 original study, the survey included multiple open-ended questions that revealed trends and patterns, allowing for a richer qualitative insight. It is important to note that the surveys were sometimes conducted in non-confidential settings, such as places of business, and other times, in the presence of friends, family and even customers. In these cases, the environment may have been distracting and could have influenced the respondents’ willingness to provide candid answers to sensitive questions. While we ensured to collect responses from multiple communities to improve representativeness, the convenience sampling approach that was taken increases the chances of selection bias (e.g. those who were willing to respond or were in the same environment might have had similar opinions) and motivational bias (e.g. respondents who were willing to partake in the survey might have been more opinionated or more willing to share their views), therefore serving as a limitation. Additionally, it was necessary to interpret the survey into local dialects for many respondents, which could have introduced bias and variation between local language dialects. Moreover, the way questions were framed during the interview process could also have influenced the responses that were given by the respondents. Furthermore, this study may not be generalisable to the other 15 regions of Ghana, as the cultural, economic and social contexts of Atwima Nwabiagya North District may be unique. For example, the Atwima Nwabiagya North District ranks 48th out of 261 districts in multidimensional poverty, indicating that it has lower poverty rates than many districts.^18^ It also has diversity in its makeup, housing both rural and urban populations.

The study results and analysis do not include tests of significance; hence, we can only describe patterns rather than causality. Specifically, we highlighted directional changes in attitudes and behaviours because we cannot control for differences between the original study’s population makeup and this follow-up study’s population. Because of the lack of access to the raw dataset from the original study, we are unable to stratify participants or adjust for potential confounders, which limits our ability to perform statistical tests. However, we did include confidence intervals for all proportional data to estimate the true proportion of the population in this most recent study.

Finally, the scope of this study was limited to a women’s-only perspective, which can potentially limit our understanding regarding contraceptives in the community. Most women reported that family planning is a joint decision between them and their partners, and most disagreed that men shouldn’t have a say in their choice of contraceptive. Therefore, understanding men’s perspectives on contraceptive use would provide more context to our results, as it is likely that their beliefs influence behaviours surrounding contraceptive use. In the future, investigating men’s beliefs and attitudes surrounding family planning would be beneficial.

## Conclusion

Multiple studies have repeatedly documented an extensive unmet need for contraception in multiple regions of Ghana,^11,19^ and our study reaffirms this. The high incidence of unplanned pregnancies, coupled with limited knowledge of contraceptive side effects, underscores the urgent need for comprehensive family planning education in the Kumasi community. Furthermore, increased reliance on emergency contraception in place of oral contraception indicates resistance to more preventative family planning measures. This seems to conflict with the reported positive attitudes surrounding the health and safety value of family planning.

Many participants indicated their willingness to learn the methods we informed them about, which is a promising sign on the path to addressing the unmet need for family planning. Going forward, it is essential to promptly implement viable methods to solve this ongoing problem.
